# Association between metabolic health indicators and cardiorespiratory fitness in urban young and middle-aged population: a retrospective cross-sectional study

**DOI:** 10.3389/fendo.2025.1489152

**Published:** 2025-05-21

**Authors:** Chengshuo Wang, Nan Yang, Qin Zhang, Ying Li, Linli Zhang, Zejian Liu, Ruoxuan Zhao, Jingman Qi, Aomeng Xiang, Yanxin Fu, Zheyu Xiong, Liang Wu, Jie Sun, Dan Huang

**Affiliations:** ^1^ Beijing Xiaotangshan Hospital, Beijing, China; ^2^ Tianjin Key Laboratory of Exercise Physiology and Sports Medicine, Institute of Sport, Exercise and Health, Tianjin University of Sport, Tianjin, China

**Keywords:** cardiorespiratory fitness, cardiopulmonary exercise testing, metabolic health indicators, urban population, cross-sectional study

## Abstract

**Background:**

Cardiorespiratory fitness (CRF) is an established risk factor for cardiovascular diseases (CVD). Compared with traditional clinical risk factors for CVD, CRF can better predict health status and possible adverse events. However, few studies have reported the association between multiple metabolic health indicators and CRF as an indicator of CVD risk. Therefore, this study aims to further understand the association between metabolic health indicators and CRF and to provide a theoretical basis for improving the early prevention strategies of CVD in the urban young and middle-aged population.

**Methods:**

A retrospective cross-sectional study was conducted on 889 young and middle-aged urban people who underwent health examinations in Beijing Xiaotangshan Hospital from January 2022 to December 2024. Baseline measurements of physical examination, biochemical examination, and cardiopulmonary exercise testing were obtained. The association between metabolic health indicators and CRF was analyzed. A multiple linear regression analysis was performed to assess the association between each metabolic health indicator and CRF and determine which metabolic health indicators may serve as useful predictors for assessing CRF.

**Results:**

We investigated the association between metabolic health indicators and CRF by adjusting for covariates (age, smoking status, and drinking status) associated with CRF. In multiple linear regression analysis, waist circumference (WC) (*β* = −0.196, *P* = 0.010), fasting plasma glucose (FPG) (*β* = −0.143, *P* < 0.001), and high-density lipoprotein cholesterol (HDL-C) (*β* = −0.125, *P* = 0.005) were significantly associated with VO_2peak_ in young and middle-aged urban men. WC (*β* = −0.577, *P* < 0.001) and FPG (*β* = −0.167, *P* = 0.002) were significantly associated with VO_2peak_ in young and middle-aged urban women. In addition, WC (men: *β* = −0.238, *P* = 0.003; women: *β* = −0.410, *P* < 0.001) and FPG (men: *β* = −0.147, *P* < 0.001; women: *β* = −0.123, *P* = 0.034) were significantly associated with AT in men and women.

**Conclusion:**

Our results showed that WC and FPG were significantly associated with CRF in young and middle-aged urban men and women. This suggests that WC and FPG may serve as useful predictors for assessing CRF within this population.

## Introduction

Cardiorespiratory fitness (CRF), also known as cardiorespiratory endurance, refers to the ability of the circulatory system and respiratory system to supply oxygen to skeletal muscles during sustained physical activity, which is a comprehensive reflection of the body’s exercise capacity (1). CRF is not only an established risk factor for cardiovascular disease (CVD) but also one of the stronger, or perhaps strongest, predictors of prognosis in the overall population and in many groups with specific CVD risk factors or established CVD conditions (2). Studies have shown that low CRF is independently associated with a 2- to 4-fold increased risk of cardiovascular disease events even after adjustment for age, BMI, blood pressure, lipid profiles, and blood glucose levels, and its predictive power surpasses that of most traditional clinical risk factors for CVD (3, 4). It is more and more often underlined that CRF assessment should be included as part of routine diagnostic procedures in all health centers (5). Unfortunately, there has been a marked decline in CRF in adults since 1980, with the magnitude of the decline gradually increasing over time. Declines were larger for men than for women and for young adults (< 40 years) than for middle-aged adults (≥ 40 years), suggestive of a corresponding decline in population health (6).

Cardiopulmonary exercise testing (CPET) is an objective, quantitative, and non-invasive CRF assessment method (7). It can integrate and analyze the continuous dynamic changes of the respiratory, circulatory, blood, and metabolic system functions during exercise to assess overall health status and CVD risk (8). Important indicators such as peak oxygen uptake (VO_2peak_) and anaerobic threshold (AT) are derived from CPET. Specifically, measuring VO_2peak_ (ml/min/kg) by CPET in the laboratory is the gold standard for objectively measuring CRF (9). VO_2peak_ can reflect the upper limit of the overall function of the cardiopulmonary system and is a strong predictor of CVD mortality, while AT can reveal the dynamic balance between metabolic efficiency and cardiovascular health, offering targeted insights for early risk prediction and rehabilitation guidance. The combination of the two can more comprehensively assess CRF and CVD risk. Although CPET is the only method that can accurately measure VO_2peak_ and AT, its popularity is low due to the need for professional equipment and certified medical personnel, the time required for a single test of 30 to 60 minutes, and the fact that it is performed at only a few medical centers (10). Therefore, finding a simpler method to estimate the VO_2peak_ and AT is of great significance for the indirect assessment of CRF.

Several studies have found that low CRF is closely related to metabolic diseases (11–14), such as obesity, diabetes, hypertension, and other diseases. However, these studies are mostly limited to observing the effect of CRF on a certain metabolic disease, and few studies have explored which metabolic health indicators may serve as useful predictors for assessing CRF. So far, CVD remains the leading cause of death worldwide (15), and the prevention of CVD is considered a global public health priority (2). Therefore, our goal in this study was to analyze the association between metabolic health indicators and CRF in the urban young and middle-aged population and to provide a theoretical basis for improving the early prevention strategies of cardiovascular diseases in this population.

## Methods

### Participants

We conducted a retrospective cross-sectional study based on data obtained from medical records. We included young and middle-aged people aged 18–59 living in the Beijing urban area who underwent health examinations (including physical examination, biochemical examination, and CPET) in Beijing Xiaotangshan Hospital from January 2022 to December 2024. A total of 58 people were excluded based on the following criteria: (a) body mass index (BMI) < 18.5 kg/m^2^; (b) missing physical examination component data; (c) missing biochemical examination component data; (d) missing CPET component data; (e) unable to cooperate with health examination and sample collection. Ultimately, 889 participants (559 men and 330 women) were included in the study. G*power 3.1.9.7 was used for *post-hoc* power analysis of the multiple linear regression model, and all results showed power ≥ 0.8, indicating that the sample size was appropriate and the multiple linear regression model had sufficient statistical power (**Supplementary Figure 1**).

Throughout the study, we did not impose any interventions on participants and did not adversely affect their rights and health. At the same time, we always ensured that participants’ privacy and personal information were fully protected. Protocols used in this study were in accordance with International Ethical Guidelines (according to the Declaration of Helsinki) and approved by the Ethics Committee of Beijing Xiaotangshan Hospital (Approval No. 2024–24). All methods were carried out in accordance with relevant guidelines and regulations. In this study, patient consent was not required as it was approved with a waiver.

### Anthropometric measurements

The general information of the participants was collected through a face-to-face diagnostic interview, including age, gender, marital status, smoking status, drinking status, and disease history. Weight and height were measured using a calibrated electronic scale while the participants were shoeless and wearing light clothing. BMI (kg/m^2^) was calculated as weight (kg) divided by height squared (m^2^). Waist circumference (WC) was taken midway between the lowest rib margin and the highest iliac crest at the end of expiration, and the maximum circumference around the buttocks while wearing thin clothing was taken as hip circumference (HC). Systolic blood pressure (SBP) and diastolic blood pressure (DBP) in both upper limbs of seated participants were measured using an Omron electronic sphygmomanometer (Omron Healthcare Co. Ltd., Kyoto, Japan), with the higher side as the measurement result, and heart rate (HR) was measured at the same time. The mean of two measurements separated by a 5-minute interval was taken as a valid determination of SBP, DBP, and HR.

### Biochemical examination

Venous blood samples were obtained from the antecubital vein after an overnight fasting period of at least eight hours to measure fasting plasma glucose (FPG), total cholesterol (TC), triglyceride (TG), high-density lipoprotein cholesterol (HDL-C), and low-density lipoprotein cholesterol (LDL-C). These assays were performed using an automatic biochemistry analyzer (Roche Cobas C 710, supplied by Beijing Barry Medical Equipment Co., Ltd., Beijing, China) according to the manufacturer’s instructions.

### Cardiorespiratory fitness

We used the Quark PFT Ergo cardiopulmonary exercise testing system produced by COSMED, Italy, to perform CPET to assess CRF. Participants tried to avoid alcohol, tea, or caffeinated beverages for three days before the test. Before the test, the tester had to ask the participants to sign an informed consent form, which included informing them of the purpose, implementation process, and precautions of CPET and reminding them of the potential discomfort and risks related to exercise. Before the test, the instrument’s gas, capacity, and flow rate were calibrated. Then, the testers transferred the participants safely to the cycle ergometer, adjusted the height suitable for pedaling, connected to the 12-lead electrocardiogram, and placed the cuff on the participants for automatic blood pressure detection and the mask for respiratory gas analysis. The tester set the power increment rate of the cycle ergometer according to the participant’s age, gender, and estimated functional status so that they could reach the symptom-limiting maximum extreme exercise within 6 to 10 minutes. During the whole exercise process, the testers closely monitored the status of the participants (16). Finally, we selected peak oxygen uptake (VO_2peak_) and anaerobic threshold (AT) as our research indicators.

### Metabolic syndrome and its components

The diagnosis of metabolic syndrome (MS) and its components is based on the diagnostic criteria of the guideline for the prevention and treatment of type 2 diabetes mellitus in China (2020 edition) (17). Three or more of the following five components can be diagnosed (1): Abdominal obesity: male WC ≥ 90 cm; female WC ≥ 85 cm; (2) Hyperglycemia: FPG ≥ 6.1 mmol/L or 2-hour postprandial blood glucose ≥ 7.8 mmol/L and/or diabetes has been diagnosed and treated; (3) Hypertension: blood pressure ≥ 130/85 mmHg (1 mmHg = 0.133 kPa) and (or) hypertension has been confirmed and treated; (4) Fasting TG ≥ 1.70 mmol/L; and (5) Fasting HDL-C < 1.04 mmol/L.

### Statistical analysis

Shapiro-Wilk test and histograms were used to detect the types of distributions of continuous variables. Continuous variables conforming to the normal distribution were described as mean ± standard deviation (SD), otherwise described as median and interquartile range. Categorical variables were described as frequencies (proportions). For continuous variables with a normal distribution, one-way ANOVA was used to compare multiple groups. For continuous variables with a skewed distribution, the Kruskal-Wallis H test was used to compare multiple groups. To correct for multiple testing, the Bonferroni correction was used. The chi-square test was used to compare the categorical variables. The Pearson or Spearman correlation coefficients were calculated to determine the association between metabolic health indicators and CRF. Multiple linear regression models were used to estimate the beta coefficients (*β*) of different metabolic health indicators on the VO_2peak_ values. Different models were applied: Model I was crude without any adjustment, and Model II was adjusted for age, smoking status, and drinking status. The collinearity of the variables in the models was assessed by calculating the variance inflation factor (VIF). Considering the difference between metabolic health indicators and CRF in men and women, the participants were divided into two groups according to sex, and the data were analyzed separately. Statistical analyses were performed using SPSS 27.0 (IBM Corp., Armonk, NY, USA). Differences were considered statistically significant at *P* < 0.05.

## Results

Retrospective cross-sectional data were available for 889 participants. The participants consisted of 559 men (62.88%) and 330 women (37.12%), with a mean age of 42.17 years (23–56 years). Regarding metabolic health indicators, SBP, DBP, BMI, WC, FPG, TG, HDL-C, and LDL-C significantly differed between men and women (*P* < 0.05). Regarding CRF, VO_2peak_ was significantly higher in men than women (*P* < 0.001). In terms of health-related behaviors, smoking and drinking rates were significantly higher in men than women (*P* < 0.001). The demographic and clinical characteristics of the study participants were summarized in **Table 1**.

**Table 1:** Demographic and clinical characteristics of study participants stratified by gender.

Variable	Total (n=889)	Male (n=559)	Female (n=330)	t/Z/χ^2^-value	*P*-value
Age (years)	43.00 (38.00, 47.00)	43.00 (39.00, 48.00)	42.00 (37.00, 46.00)	−3.911	< 0.001
Height (cm)	169.50 (163.00, 175.00)	173.5 (169.50, 177.00)	161.50 (158.00, 164.50)	−21.397	< 0.001
Weight (kg)	72.00 (61.60, 81.10)	77.00 (70.5, 84.70)	59.80 (54.40, 70.10)	−16.609	< 0.001
HR (bpm)	84.00 (74.00, 93.00)	83.00 (74.00, 91.00)	85.00 (77.00, 94.00)	−2.775	0.006
SBP (mmHg)	120.00 (111.50, 130.00)	125.00 (115.00, 133.00)	115.00 (107.00, 123.00)	−9.486	< 0.001
DPB (mmHg)	73.00 (66.50, 82.00)	76.00 (70.00, 83.00)	70.00 (64.00, 75.25)	−8.309	< 0.001
BMI (kg/m^2^)	24.92 (22.59, 27.38)	25.79 (23.76, 27.80)	23.12 (21.01, 26.21)	−8.891	< 0.001
WC (cm)	85.00 (76.00, 91.00)	88.00 (83.00, 94.00)	75.00 (69.00, 82.00)	−17.079	< 0.001
HC (cm)	97.00 (93.00, 101.00)	98.00 (95.00, 102.00)	95.00 (91.00, 100.00)	−7.974	< 0.001
FPG (mmol/L)	5.10 (4.77, 5.54)	5.17 (4.82, 5.67)	4.94 (4.67, 5.29)	−5.889	< 0.001
TC (mmol/L)	4.86 (4.34, 5.43)	4.89 (4.35, 5.48)	4.79 (4.34, 5.33)	−0.937	0.349
TG (mmol/L)	1.23 (0.91, 1.90)	1.45 (1.05, 2.21)	0.97 (0.71, 1.30)	−11.882	< 0.001
HDL-C (mmol/L)	1.33 (1.12, 1.58)	1.22 (1.05, 1.41)	1.55 (1.33, 1.73)	−12.535	< 0.001
LDL-C (mmol/L)	3.22 (2.73, 3.85)	3.37 (2.76, 3.92)	3.14 (2.67, 3.74)	−2.516	0.012
VO_2peak_ (ml/min/kg)	23.10 (20.10, 25.95)	21.50 (18.58, 24.03)	21.80 (19.60, 23.30)	−8.068	< 0.001
AT (ml/min/kg)	13.10 (11.50, 14.80)	12.20 (11.10, 13.30)	13.10 (11.20, 15.30)	−0.030	0.976
Smoking, n (%)	194 (21.82)	187 (33.45)	7 (2.12)	120.053	< 0.001
Drinking, n (%)	391 (43.98)	334 (59.75)	57 (17.27)	153.290	< 0.001

Continuous variables are expressed as mean ± standard deviation (SD) or median (interquartile range). Categorical variables are expressed as the number and percentage as indicated in the table. HR, heart rate; SBP, systolic blood pressure; DBP, diastolic blood pressure; BMI, body mass index; WC, waist circumference; HC, hip circumference; FPG, fasting plasma glucose; TC, total cholesterol; TG, triglyceride; HDL-C, high-density lipoprotein cholesterol; LDL-C, low-density lipoprotein cholesterol; VO_2peak_, peak oxygen uptake; AT, anaerobic threshold.

**Figure 1:**
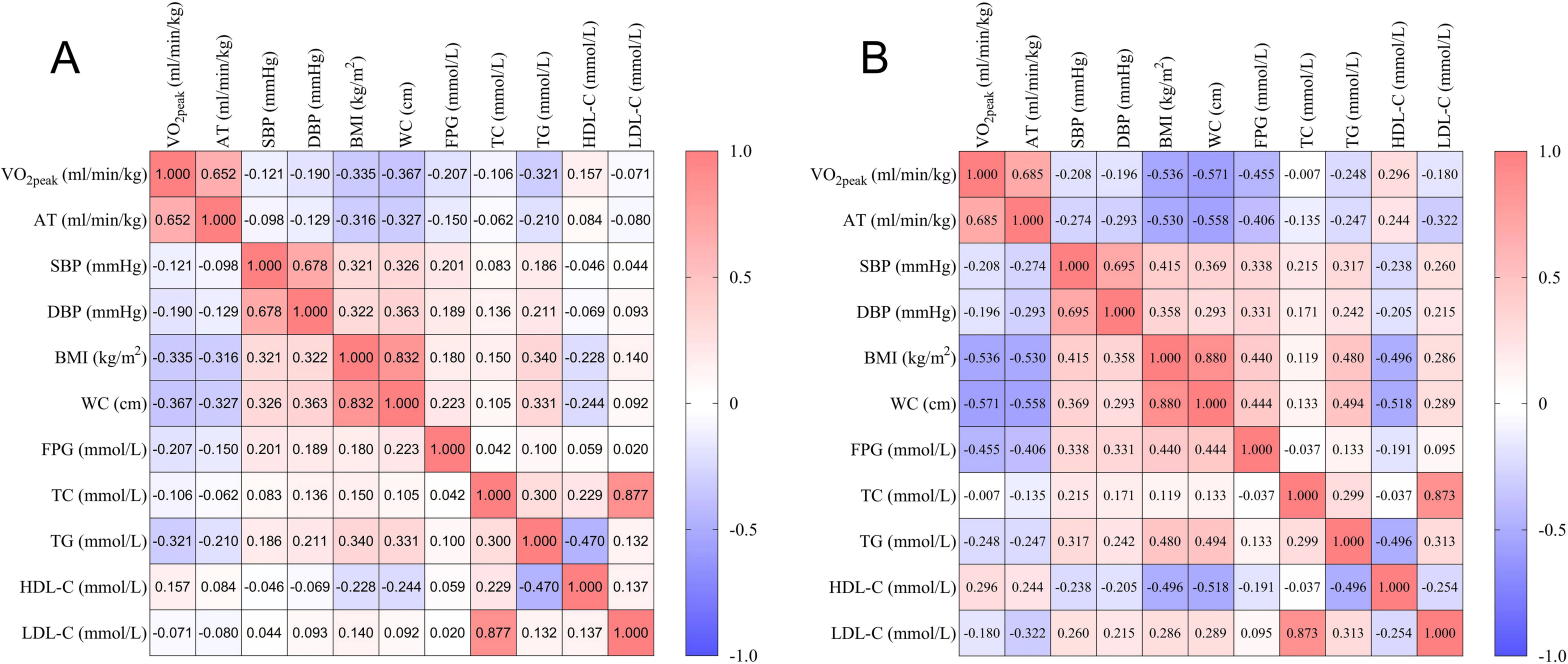
Heat map of correlation analysis. **(A)**, Male; **(B)**, Female.

The Pearson or Spearman correlation coefficient was calculated to analyze the association between metabolic health indicators and CRF (**Table 2**, **Figure 1**). In males, VO_2peak_ was negatively correlated with SBP, DBP, BMI, WC, FPG, TC, and TG (*P* < 0.05), and positively correlated with HDL-C (*P* < 0.05). AT was negatively correlated with SBP, DBP, BMI, WC, FPG, and TG (*P* < 0.05), and positively correlated with HDL-C (*P* < 0.05). In females, VO_2peak_ was negatively correlated with SBP, DBP, BMI, WC, FPG, TG, and LDL-C (*P* < 0.05), and positively correlated with HDL-C (*P* < 0.05). AT was negatively correlated with SBP, DBP, BMI, WC, FPG, TC, TG, and LDL-C (*P* < 0.05), and positively correlated with HDL-C (*P* < 0.05).

**Table 2:** Correlations of the CRF with the metabolic health indicators by gender.

Variable	Male (n=559)				Female (n=330)			
	VO_2peak_ (ml/min/kg)		AT (ml/min/kg)		VO_2peak_ (ml/min/kg)		AT (ml/min/kg)	
	*r*	*P*	*r*	*P*	*r*	*P*	*r*	*P*
SBP (mmHg)	−0.121	0.004	−0.098	0.020	−0.208	< 0.001	−0.247	< 0.001
DBP (mmHg)	−0.190	< 0.001	−0.129	0.002	−0.196	< 0.001	−0.293	< 0.001
BMI (kg/m^2^)	−0.335	< 0.001	−0.316	< 0.001	−0.536	< 0.001	−0.530	< 0.001
WC (cm)	−0.367	< 0.001	−0.327	< 0.001	−0.571	< 0.001	−0.558	< 0.001
FPG (mmol/L)	−0.207	< 0.001	−0.150	< 0.001	−0.455	< 0.001	−0.406	< 0.001
TC (mmol/L)	−0.106	0.012	−0.062	0.142	−0.007	0.905	−0.135	0.014
TG (mmol/L)	−0.321	< 0.001	−0.210	< 0.001	−0.248	< 0.001	−0.247	< 0.001
HDL-C (mmol/L)	0.157	< 0.001	0.084	0.046	0.296	< 0.001	0.244	< 0.001
LDL-C (mmol/L)	−0.071	0.094	−0.080	0.059	−0.180	0.001	−0.322	< 0.001

r, correlation value; SBP, systolic blood pressure; DBP, diastolic blood pressure; BMI, body mass index; WC, waist circumference; FPG, fasting plasma glucose; TC, total cholesterol; TG, triglyceride; HDL-C, high-density lipoprotein cholesterol; LDL-C, low-density lipoprotein cholesterol; VO_2peak_, peak oxygen uptake; AT, anaerobic threshold.

In the multiple linear regression model, VO_2peak_ and AT were used as dependent variables, and metabolic health indicators were used as independent variables. Variables with no correlation or with multicollinearity were not included in the regression model. Finally, we analyzed the predictive effects of SBP, DBP, BMI, WC, FPG, TC, TG, and HDL-C on VO_2peak_ and AT in males and females, respectively (**Tables 3**, **4**). Model I was crude without any adjustment, and Model II was adjusted for age, smoking status, and drinking status.

**Table 3:** Association between metabolic health indicators and VO_2peak_ by gender.

Variable	Model I				Model II			
	B	SE	*β* (95% CI)	*P*-value	B	SE	*β* (95% CI)	*P*-value
Male (n=559)								
SBP (mmHg)	0.027	0.018	0.083 (−0.007 to 0.062)	0.121	0.022	0.017	0.067 (−0.012 to 0.056)	0.211
DBP (mmHg)	−0.040	0.024	−0.090 (−0.087 to 0.007)	0.096	−0.029	0.024	−0.065 (−0.076 to 0.019)	0.233
BMI (kg/m^2^)	−0.099	0.111	−0.067 (−0.318 to 0.120)	0.374	−0.132	0.111	0.089 (−0.350 to 0.086)	0.234
WC (cm)	−0.128	0.043	−0.227 (−0.212 to −0.044)	0.003	−0.111	0.043	−0.196 (−0.195 to −0.026)	0.010
FPG (mmol/L)	−0.798	0.199	−0.161 (−1.189 to −0.406)	< 0.001	−0.709	0.200	−0.143 (−1.101 to −0.317)	< 0.001
TC (mmol/L)	−0.200	0.204	−0.042 (−0.601 to 0.201)	0.328	−0.199	0.202	−0.042 (−0.597 to 0.199)	0.326
TG (mmol/L)	−0.152	0.102	−0.065 (−0.352 to 0.048)	0.137	−0.149	0.101	−0.064 (−0.347 to 0.050)	0.141
HDL-C (mmol/L)	1.538	0.637	0.108 (0.287 to 2.790)	0.016	1.789	0.636	0.125 (0.539 to 3.039)	0.005
Female (n=330)								
SBP (mmHg)	0.035	0.019	0.115 (−0.004 to 0.073)	0.076	0.035	0.019	0.115 (−0.004 to 0.073)	0.076
DBP (mmHg)	−0.016	0.025	−0.040 (−0.066 to 0.033)	0.517	−0.015	0.025	−0.036 (−0.064 to 0.035)	0.559
BMI (kg/m^2^)	−0.004	0.106	−0.004 (−0.213 to 0.205)	0.971	−0.022	0.107	−0.020 (−0.233 to 0.189)	0.839
WC (cm)	−0.255	0.043	−0.592 (−0.340 to −0.171)	< 0.001	−0.249	0.043	−0.577 (−0.334 to −0.164)	< 0.001
FPG (mmol/L)	−1.345	0.389	−0.181 (−2.110 to −0.580)	0.001	−1.243	0.398	−0.167 (−2.026 to −0.459)	0.002
TC (mmol/L)	0.097	0.253	0.018 (−0.402 to 0.596)	0.702	0.121	0.254	0.022 (−0.379 to 0.621)	0.633
TG (mmol/L)	0.288	0.371	0.041 (−0.441 to 1.018)	0.437	0.366	0.376	0.052 (−0.375 to 1.106)	0.332
HDL-C (mmol/L)	−0.346	0.653	−0.029 (−1.631 to 0.939)	0.597	−0.214	0.662	−0.018 (−1.517 to 1.089)	0.747

Model I, crude model; Model II, adjusted for age, smoking status, and drinking status; B, unstandardized coefficient; SE, standard error; *β*, standardized regression coefficient; SBP, systolic blood pressure; DBP, diastolic blood pressure; BMI, body mass index; WC, waist circumference; FPG, fasting plasma glucose; TC, total cholesterol; TG, triglyceride; HDL-C, high-density lipoprotein cholesterol; VO_2peak_, peak oxygen uptake; AT, anaerobic threshold.

**Table 4:** Association between metabolic health indicators and AT by gender.

Variable	Model I				Model II			
	B	SE	*β* (95% CI)	*P*-value	B	SE	*β* (95% CI)	*P*-value
Male (n=559)								
SBP (mmHg)	0.014	0.010	0.077 (−0.006 to 0.034)	0.167	0.015	0.010	0.082 (−0.005 to 0.036)	0.139
DBP (mmHg)	−0.015	0.014	−0.059 (−0.043 to 0.013)	0.295	−0.017	0.014	−0.068 (−0.045 to 0.011)	0.231
BMI (kg/m^2^)	−0.063	0.065	−0.075 (−0.191 to 0.065)	0.336	−0.056	0.066	−0.067 (−0.185 to 0.073)	0.391
WC (cm)	−0.073	0.025	−0.227 (−0.122 to −0.023)	0.004	−0.076	0.025	−0.238 (−0.126 to −0.026)	0.003
FPG (mmol/L)	−0.397	0.117	−0.140 (−0.627 to −0.167)	0.001	−0.415	0.118	−0.147 (−0.647 to −0.183)	< 0.001
TC (mmol/L)	−0.009	0.120	−0.003 (−0.245 to 0.226)	0.939	−0.009	0.120	−0.003 (−0.245 to 0.226)	0.938
TG (mmol/L)	−0.009	0.060	−0.007 (−0.127 to 0.108)	0.876	−0.010	0.060	−0.007 (−0.127 to 0.107)	0.868
HDL-C (mmol/L)	0.421	0.374	0.052 (−0.314 to 1.156)	0.261	0.370	0.377	0.046 (−0.370 to 1.110)	0.326
Female (n=330)								
SBP (mmHg)	0.016	0.014	0.077 (−0.012 to 0.044)	0.268	0.016	0.014	0.077 (−0.012 to 0.044)	0.268
DBP (mmHg)	−0.034	0.018	−0.123 (−0.070 to 0.003)	0.069	−0.033	0.018	−0.120 (−0.069 to 0.004)	0.078
BMI (kg/m^2^)	−0.056	0.078	−0.078 (−0.210 to 0.097)	0.471	−0.067	0.079	−0.093 (−0.223 to 0.088)	0.394
WC (cm)	−0.124	0.032	−0.423 (−0.186 to −0.062)	< 0.001	−0.120	0.032	−0.410 (−0.183 to −0.057)	< 0.001
FPG (mmol/L)	−0.687	0.286	−0.136 (−1.250 to −0.124)	0.017	−0.624	0.293	−0.123 (−1.200 to −0.047)	0.034
TC (mmol/L)	−0.260	0.186	−0.069 (−0.627 to 0.107)	0.164	−0.245	0.187	−0.065 (−0.613 to 0.123)	0.191
TG (mmol/L)	−0.093	0.273	−0.019 (−0.630 to 0.443)	0.733	−0.045	0.277	−0.009 (−0.590 to 0.500)	0.870
HDL-C (mmol/L)	−0.830	0.481	−0.101 (−1.776 to 0.115)	0.085	−0.749	0.488	−0.091 (−1.708 to 0.211)	0.126

Model I, crude model; Model II, adjusted for age, smoking status, and drinking status; B, unstandardized coefficient; SE, standard error; *β*, standardized regression coefficient; SBP, systolic blood pressure; DBP, diastolic blood pressure; BMI, body mass index; WC, waist circumference; FPG, fasting plasma glucose; TC, total cholesterol; TG, triglyceride; HDL-C, high-density lipoprotein cholesterol; VO_2peak_, peak oxygen uptake; AT, anaerobic threshold.

In the male-adjusted model with VO_2peak_ as the dependent variable, the P-P plot showed that the residuals were approximately normally distributed, the degree of multicollinearity among the variables was low (all VIFs < 5; Tolerance > 0.2) (**Supplementary Table 1**), and the multiple linear regression model was statistically significant (adjusted R^2^ = 0.187, F = 15.238, *P* < 0.001). Among the variables included in the model, WC (*β* = −0.196, 95% CI: −0.195 to −0.026, *P* = 0.010), FPG (*β* = −0.143, 95% CI: −1.101 to −0.317, *P* < 0.001), and HDL-C (*β* = 0.125, 95% CI: 0.539 to 3.039, *P* = 0.005) had statistically significant effects on VO_2peak_. In the male-adjusted model with AT as the dependent variable, the P-P plot showed that the residuals were approximately normally distributed, the degree of multicollinearity among the variables was low (all VIFs < 5; Tolerance > 0.2) (**Supplementary Table 2**), and the multiple linear regression model was statistically significant (adjusted R^2^ = 0.122, F = 9.605, *P* < 0.001). Among the variables included in the model, WC (*β* = −0.238, 95% CI: −0.126 to −0.026, *P* = 0.003) and FPG (*β* = −0.147, 95% CI: −0.647 to −0.183, *P* < 0.001) had statistically significant effects on AT.

In the female-adjusted model with VO_2peak_ as the dependent variable, the P-P plot showed that the residuals were approximately normally distributed, the degree of multicollinearity among the variables was low (all VIFs < 6; Tolerance > 0.2) (**Supplementary Table 1**), and the multiple linear regression model was statistically significant (adjusted R^2^ = 0.412, F = 26.612, *P* < 0.001). Among the variables included in the model, WC (*β* = −0.577, 95% CI: −0.334 to −0.164, *P* < 0.001) and FPG (*β* = −0.167, 95% CI: −2.026 to −0.459, *P* = 0.002) had statistically significant effects on VO_2peak_. In the female-adjusted model with AT as the dependent variable, the P-P plot showed that the residuals were approximately normally distributed, the degree of multicollinearity among the variables was low (all VIFs < 6; Tolerance > 0.2) (**Supplementary Table 2**), and the multiple linear regression model was statistically significant (adjusted R^2^ = 0.310, F = 17.399, *P* < 0.001). Among the variables included in the model, WC (*β* = −0.410, 95% CI: −0.183 to −0.057, *P* < 0.001) and FPG (*β* = −0.123, 95% CI: −1.200 to −0.047, *P* = 0.034) had statistically significant effects on AT.

**Figure 2:**
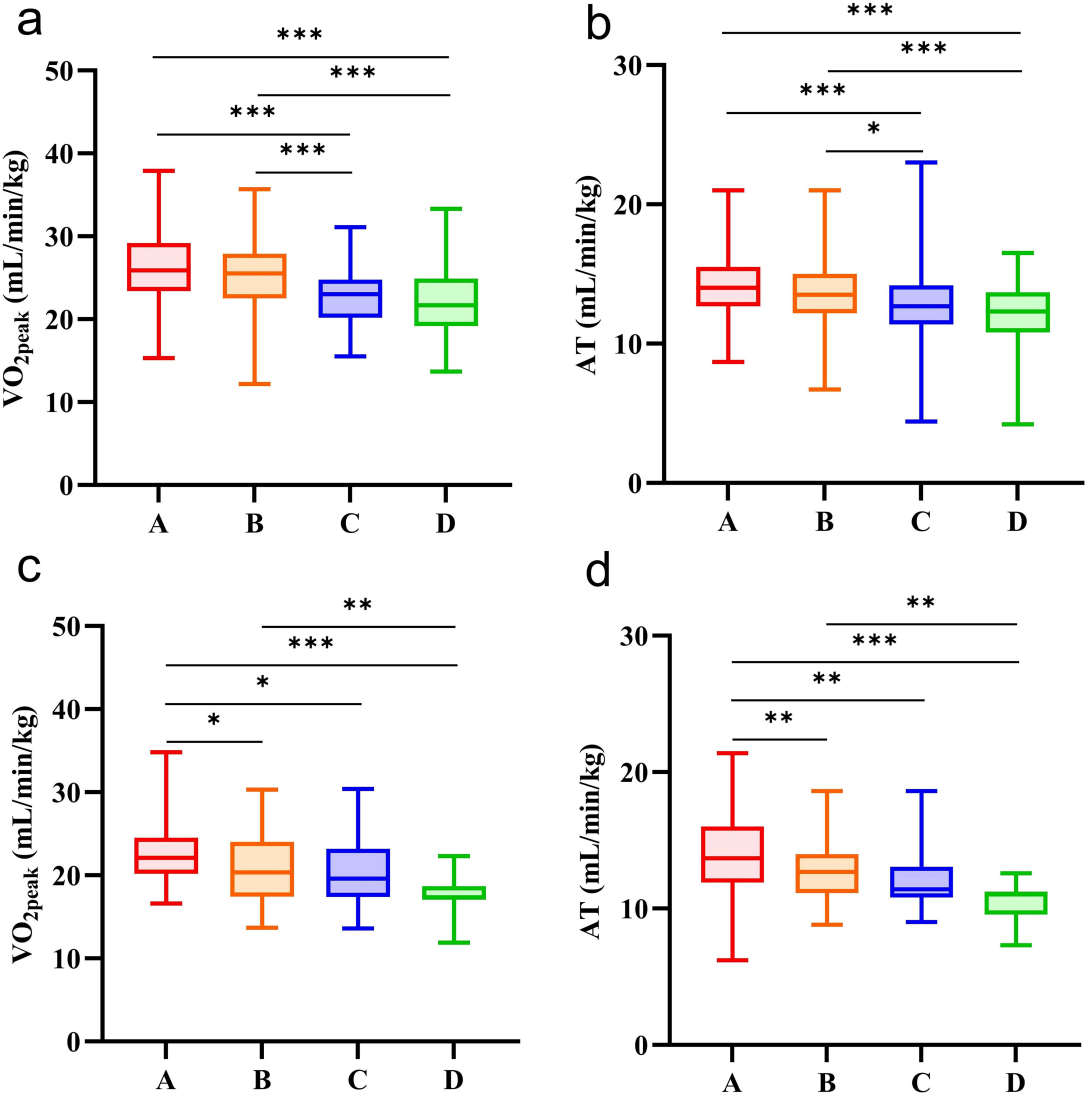
CRF in participants with different numbers of MS components. **(a)** comparison of VO_2peak_ in male participants with different numbers of MS components. **(b)** comparison of AT in male participants with different numbers of MS components. **(c)** comparison of VO_2peak_ in female participants with different numbers of MS components. d, comparison of AT in female participants with different numbers of MS components. **A** the number of MS components (n = 0); **B** the number of MS components (n = 1); **C** the number of MS components (n = 2); **D** the number of MS components (n ≥ 3). *, the comparison between the two groups was statistically significant (Adj. *P* < 0.05). **the comparison between the two groups was statistically significant (Adj. *P* < 0.01). ***the comparison between the two groups was statistically significant (Adj. *P* < 0.001).

In this study, the Kruskal-Wallis H test was used to analyze the influence of the number of MS components on CRF in the urban young and middle-aged population (**Table 5**, **Figure 2**), and Bonferroni correction was used for pairwise comparison between the two groups (**Supplementary Figure 2**). In males, participants with 2 or ≥ 3 MS components had lower VO_2peak_ and AT than those with 1 or 0 MS components (Adj. *P* < 0.05). In females, participants with 1, 2 or ≥ 3 MS components had lower VO_2peak_ and AT than those with 0 MS components (Adj. *P* < 0.05). When the number of MS components was ≥ 3, VO_2peak_ and AT were lower than those in participants with 1 MS component (Adj. *P* < 0.05).

**Table 5:** CRF in participants with different numbers of MS components.

Variable	The number of MS components (n = 0)	The number of MS components (n = 1)	The number of MS components (n = 2)	The number of MS components (n ≥ 3)	Z-value	*P*-value
Male (n=559)						
VO_2peak_ (ml/min/kg)	26.00 (23.00, 29.20)	24.80 (22.15, 27.40)	22.85 (20.13, 25.58)^ab^	22.20 (19.50, 25.00)^ab^	78.803	< 0.001
AT (ml/min/kg)	14.00 (12.63, 16.00)	13.40 (12.15, 14.80)	12.10 (10.90, 14.18)^ab^	12.80 (11.10, 14.10)^ab^	56.200	< 0.001
Female (n=330)						
VO_2peak_ (ml/min/kg)	22.20 (20.30, 24.30)	20.40 (17.50, 24.00)^a^	18.70 (16.83, 21.55)^a^	17.25 (13.20, 18.55)^ab^	38.364	< 0.001
AT (ml/min/kg)	13.40 (11.80, 15.90)	13.30 (11.30, 14.70)^a^	11.15 (10.78, 12.20)^a^	10.80 (8.98, 11.13)^ab^	47.057	< 0.001

MS, metabolic syndrome; VO_2peak_, peak oxygen uptake; AT, anaerobic threshold. ^a^significantly different from the number of MS components (n = 0) (Adj. *P* < 0.05). ^b^significantly different from the number of MS components (n = 1) (Adj. *P* < 0.05). ^c^significantly different from the number of MS components (n = 2) (Adj. *P* < 0.05).

## Discussion

Low CRF in adults is associated with greater risks for congestive heart failure, stroke, type 2 diabetes mellitus, some cancers, and neuropsychological disturbances (e.g., dementia, anxiety, and depression) (18, 19). Most importantly, improvements in CRF over time are associated with reduced mortality and morbidity. This provides a clear signal that having higher levels of CRF is essential for maintaining population health (20). The assessment of CRF requires a standardized CPET in the laboratory. Compared with traditional metabolic indicators, CPET can provide a more comprehensive physiological function assessment and earlier risk warning. However, CPET needs to be equipped with a gas metabolism analyzer, electrocardiogram monitoring system, etc., which not only incurs high costs but also requires a strict standardization process, making it difficult to popularize in grassroots hospitals, which limits the wide application to a certain extent (16). The traditional metabolic health indicators are easy to obtain through routine physical examination, with low cost and high penetration rate (21). So far, few studies have reported the association between multiple metabolic health indicators and CRF as an indicator of CVD risk. Therefore, we bridged this gap through this study and finally found that WC and FPG may serve as useful predictors for assessing CRF in young and middle-aged urban men and women. We propose a strategy of combining metabolic health indicators with CPET: traditional indicators (such as WC and FPG) for primary screening and CPET for in-depth assessment in high-risk populations to optimize the CVD risk stratification model.

Obesity has increasingly become one of the important problems that seriously threaten human physical and mental health in the 21st century. There is a large amount of epidemiological evidence that there is a robust negative correlation between obesity and CRF, and this association still exists after adjusting for the level of physical activity (6, 22). BMI (kg/m^2^) is calculated as weight (kg) divided by height squared (m^2^), which is one of the standards commonly used in international clinical practice to measure the degree of obesity (23). However, some studies have pointed out that BMI is an imperfect measure (24), and it cannot excellently assess fat distribution and the cardiometabolic risk associated with increased obesity (25). As a special form of obesity, abdominal obesity is closely related to the morbidity and mortality risk of CVD (26). WC is a simple method to assess abdominal obesity that is easy to standardize and clinically apply (25), and to some extent, it can make up for the deficiency that BMI cannot assess fat distribution (27). A cross-sectional study of 807 men and 633 women in Finland found that individuals with the same BMI category and normal WC had better CRF than those with higher WC (22). A cross-sectional study that only included young men showed that CRF was more closely related to WC than BMI (28). This study found that WC was negatively correlated with VO_2peak_ and AT in young and middle-aged urban men and women. Multiple linear regression analysis showed that WC was one of the important negative factors of VO_2peak_ and AT in the urban young and middle-aged population. These studies have shown that WC, as a simple assessment tool in clinical practice, may serve as a useful predictor for assessing CRF levels and CVD risk.

Diabetes mellitus (DM) is a group of metabolic diseases characterized by hyperglycemia resulting from defects in insulin secretion, insulin action, or both (29). Type 2 diabetes mellitus (T2DM) is the most common DM worldwide, accounting for nearly 90% of DM prevalent cases (30). Because FPG is minimally disturbed by diet (31), it is considered an important reference tool for judging blood glucose levels and diagnosing diabetes compared with random blood glucose levels (32). This study found that FPG was negatively correlated with VO_2peak_ and AT in young and middle-aged urban men and women. Multiple linear regression analysis showed that FPG was one of the important negative factors of VO_2peak_ and AT in the urban young and middle-aged population. Recent studies have also shown that DM has a significant adverse effect on CRF. Although this effect is partially mitigated in people with high physical activity levels, it is still significant. In addition, CRF impairment is greater in patients with T2DM compared with type 1 diabetes mellitus (T1DM) (33). In patients with T2DM, CRF impairment is more significant in women than in men, with similar results in adolescents (34). Many factors, such as insulin resistance, skeletal muscle and microvascular system dysfunction, may lead to this phenomenon. As a vasoactive hormone, insulin has been shown to increase skeletal and myocardial perfusion in rodents and healthy humans (35–37), and this perfusion response is attenuated in insulin-resistant states (38, 39). In addition, studies have found that microvascular blood flow is significantly reduced in insulin-resistant animal models and human participants (40). These studies have demonstrated the role of insulin in the heart, skeletal muscle, and microvascular system, and insulin is one of the important factors affecting CRF. In addition to insulin’s role in regulating perfusion, hyperglycemia can also lead to the production of advanced glycation end products (AGEs) (41). These accrue in the wall of vessels, leading to integral loss of the structure of the vessel wall and underlying basement membrane, as well as proinflammatory signaling contributing to systemic vascular dysfunction, which may also lead to decreased CRF (42–44). To sum up, these findings show that high blood glucose can significantly reduce CRF and increase the risk of CVD, especially for people with T2DM, and also suggest that people with high blood glucose should regularly carry out exercise rehabilitation training to maintain or improve their CRF.

HDL-C is generally described as “good cholesterol”, which can participate in the reverse transport of cholesterol, has antioxidant, anti-inflammatory, and anti-thrombotic properties (45), and its anti-atherosclerotic function is vital in promoting cardiovascular health. Liu et al. (46) reported a positive association between CRF and HDL-C in men after adjustment for age, smoking status, and drinking status. Musa et al. (47) reported similar findings. In addition, HDL-C has been demonstrated to be a reliable and consistent predictor of CVD independent of other risk factors (48, 49). However, current epidemiological studies have shown that in CVD patients, the association between HDL-C concentrations and all-cause mortality exhibited a U-shaped association for both men and women, with both extremely high and low concentrations being associated with high all-cause mortality risk (50, 51). Moreover, niacin (52) and cholesteryl ester transfer protein inhibitors (53) increased HDL-C but did not show clinical benefit. This study found a positive correlation between CRF and HDL-C in urban young and middle-aged men. Further multiple linear regression analysis showed that higher levels of HDL-C were significantly associated with CRF. However, based on the above studies, we believe that the association between HDL-C and CRF should not be prematurely concluded, and further exploration is needed in the future. At the same time, we also found that HDL-C had no significant effect on VO_2peak_ in multiple linear regression analysis of young and middle-aged women in urban areas. The possible reasons include (1): Higher estrogen levels in females may partially replace the protective effect of HDL-C by enhancing vascular endothelial function and anti-inflammatory effects, thus weakening the direct association between HDL-C and VO_2peak_ (54) (2). Regarding fat distribution, compared with men, women have a higher proportion of subcutaneous fat and lower metabolic activity, which may reduce the association between HDL-C and visceral fat inflammation and weaken the contribution of HDL-C to VO_2peak_ (55) (3). Regarding HDL particle characteristics, male HDL particles are smaller and have stronger anti-inflammatory and antioxidant capacities, which directly supports the efficiency of oxygen transport during exercise, while female HDL particles exhibit the opposite characteristics (56).

The number of MS components is significant in evaluating the severity of metabolic disorders, so we analyzed the relationship between the number of MS components and CRF. The results showed that CRF decreased significantly with the increase in the number of MS components (especially when the number of male MS components was 2 or ≥ 3 or the number of female MS components was 1, 2, or ≥ 3). This result indicates that a variety of metabolic disorders may be involved in the decline of CRF, including obesity, hyperglycemia, hypertension, hyperlipidemia, etc., and these components may have a superimposed effect on each other. Particular attention should be paid when there is abdominal obesity or hyperglycemia, especially when the two coexist because we found that WC and FPG were the most significant factors affecting CRF in metabolic health indicators through multiple linear regression analysis. Therefore, the results of this study can be used to develop individualized intervention programs in clinical practice to reduce the severity of metabolic disorders and optimize CRF, thereby reducing the risk of CVD.

This study found that WC and FPG may serve as useful predictors for assessing CRF in young and middle-aged urban men and women. However, it should be emphasized that the bidirectional association between CRF and metabolic risk factors has been well established (57). The existing evidence demonstrates a dynamic interplay between the two: on the one hand, metabolic dysfunction may impair CRF through mechanisms such as insulin resistance and mitochondrial dysfunction (34); on the other hand, the improvement of CRF can improve metabolic indicators by ameliorating vascular endothelial function and augmenting skeletal muscle oxidative capacity (58). Despite this bidirectional association, our findings retain significant implications (1): In primary care, WC and FPG measurements could serve as practical screening tools for identifying individuals with low CRF, enabling early intervention (2). This study suggests that future intervention trials could target these specific metabolic health indicators for combined intervention (such as WC reduction combined with FPG control) to explore whether this method is more effective than exercise intervention alone in improving CRF (3). From a clinical point of view, the results of this study highlight the necessity of a dual targeting strategy: both using metabolic health indicators for CRF risk stratification and breaking the vicious cycle of metabolic deterioration by enhancing CRF.

The study also has some limitations. First, this is a retrospective cross-sectional study, so we only report correlations and cannot draw further conclusions about causation. Second, the number of participants in this study is small, and participants are limited to one geographic area. Therefore, the results of this study may not be generalized to rural populations or ethnically diverse groups. In the future, we need to expand the geographical area and collect more data to verify the consistency of the research conclusions in different regions. Third, as a retrospective cross-sectional study, this study failed to determine the required sample size based on *a priori* power analysis. Although the test power is evaluated by *post-hoc* power analysis, the estimation of effect size in retrospective design may be interfered with selection bias and uncontrolled confounding factors, resulting in bias in the evaluation of statistical power. Finally, the CRF of all participants was measured by a cycle ergometer, but the CRF measured by a cycle ergometer may be 5–10% lower than the CRF measured by treadmill testing (59), which makes it difficult to reflect the limit of cardiopulmonary fitness. However, the load of the cycle ergometer is controllable, the data is stable, and the applicable population is wider (such as those with insufficient motor skills, lower limb joint injury, and poor coordination), so it is the first choice.

## Conclusion

Our results showed that WC and FPG were significantly associated with CRF in young and middle-aged urban men and women. This suggests that WC and FPG may serve as useful predictors for assessing CRF within this population. In the future, further studies with a larger sample size of participants are needed to determine the validity of our findings.

## Data Availability

The original contributions presented in the study are included in the article/**Supplementary Material**. Further inquiries can be directed to the corresponding authors.

## References

[1] Raghuveer G Hartz J Lubans DR Takken T Wiltz JL Mietus-Snyder M. Cardiorespiratory fitness in youth: an important marker of health: A scientific statement from the american heart association. Circulation. (2020) 142:e101–18. doi: 10.1161/CIR.0000000000000866 PMC7524041 32686505

[2] Lavie CJ Ozemek C Carbone S Katzmarzyk PT Blair SN. Sedentary behavior, exercise, and cardiovascular health. Circ Res. (2019) 124:799–815. doi: 10.1161/CIRCRESAHA.118.312669 30817262

[3] Mandsager K Harb S Cremer P Phelan D Nissen SE Jaber W. Association of cardiorespiratory fitness with long-term mortality among adults undergoing exercise treadmill testing. JAMA Netw Open. (2018) 1:e183605. doi: 10.1001/jamanetworkopen.2018.3605 30646252 PMC6324439

[4] Myers J de Souza E Silva CG Arena R Kaminsky L Christle JW Busque V. Comparison of the FRIEND and wasserman-hansen equations in predicting outcomes in heart failure. J Am Heart Assoc. (2021) 10:e021246. doi: 10.1161/JAHA.121.021246 34689609 PMC8751827

[5] Ross R Myers J. Cardiorespiratory fitness and its place in medicine. Rev Cardiovasc Med. (2023) 24:14. doi: 10.31083/j.rcm2401014 39076861 PMC11270451

[6] Lamoureux NR Fitzgerald JS Norton KI Sabato T Tremblay MS Tomkinson GR. Temporal trends in the cardiorespiratory fitness of 2,525,827 adults between 1967 and 2016: A systematic review. Sports Med. (2019) 49:41–55. doi: 10.1007/s40279-018-1017-y 30390202

[7] Chinese Society of Cardiology Chinese Medical Association Professional Committee of Cardiopulmonary Prevention and Rehabilitation of Chinese Rehabilitation Medical Association Editorial Board of Chinese Journal of Cardiology. Chinese expert consensus on exercise rehabilitation of stab le angina pectoris. Zhonghua Xin Xue Guan Bing Za Zhi. (2023) 51:1033–42. doi: 10.3760/cma.j.cn112148-20230814-00076 37859355

[8] Herdy AH Ritt LEF Stein R de Araújo CGS Milani M RS M. Cardiopulmonary exercise test: background, applicability and interpretation. Arq Bras Cardiol. (2016) 107:467–81. doi: 10.5935/abc.20160171 PMC5137392 27982272

[9] Ross RM. ATS/ACCP statement on cardiopulmonary exercise testing. Am J Respir Crit Care Med. (2003) 167:1451. doi: 10.1164/ajrccm.167.10.950 12738602

[10] Koen M Kubota Y Tokita M Kato K Takahashi H Akutsu K. Relationship of maximum walking speed with peak oxygen uptake and anaerobic threshold in male patients with heart failure. Heart Vessels. (2023) 38:1344–55. doi: 10.1007/s00380-023-02289-y PMC10520159 37493799

[11] Zhao Y Qie R Han M Huang S Wu X Zhang Y. Independent and joint associations of non-exercise cardiorespiratory fitness and obesity with risk of type 2 diabetes mellitus in the Rural Chinese Cohort Study. Nutr Metab Cardiovasc Dis. (2022) 32:929–36. doi: 10.1016/j.numecd.2022.01.005 35067443

[12] Sloan RA Kim Y Kenyon J Visentini-Scarzanella M Sawada SS Sui X. Association between estimated cardiorespiratory fitness and abnormal glucose risk: A cohort study. J Clin Med. (2023) 12:2740. doi: 10.3390/jcm12072740 37048823 PMC10095416

[13] Zhao Y Fu X Ke Y Wu Y Qin P Hu F. Independent and joint associations of estimated cardiorespiratory fitness and its dynamic changes and obesity with the risk of hypertension: A prospective cohort. J Hum Hypertens. (2024) 38:413–9. doi: 10.1038/s41371-024-00910-9 38600254

[14] Patel PH Gates M Kokkinos P Lavie CJ Zhang J Sui X. Non-exercise estimated cardiorespiratory fitness and incident hypertension. Am J Med. (2022) 135:906–14. doi: 10.1016/j.amjmed.2022.01.048 PMC9233001 35235822

[15] Roth GA Mensah GA Johnson CO Addolorato G Ammirati E Baddour LM. Global burden of cardiovascular diseases and risk factors, 1990–2019. J Am Coll Cardiol. (2020) 76:2982–3021. doi: 10.1016/j.jacc.2020.11.010 33309175 PMC7755038

[16] Balady GJ Arena R Sietsema K Myers J Coke L Fletcher GF. Clinician’s Guide to cardiopulmonary exercise testing in adults: a scientific statement from the American Heart Association. Circulation. (2010) 122:191–225. doi: 10.1161/CIR.0b013e3181e52e69 20585013

[17] Chinese Elderly Type 2 Diabetes Prevention and Treatment of Clinical Guidelines Writing Group Geriatric Endocrinology and Metabolism Branch of Chinese Geriatric Society Geriatric Endocrinology and Metabolism Branch of Chinese Geriatric Health Care Society Geriatric Professional Committee of Beijing Medical Award Foundation National Clinical Medical Research Center for Geriatric Diseases (PLA General Hospital). Clinical guidelines for prevention and treatment of type 2 diabetes mellitus in the elderly in China (2022 edition). Zhonghua Nei Ke Za Zhi. (2022) 61:12–50. doi: 10.3760/cma.j.cn112138-20211027-00751 34979769

[18] Kodama S Saito K Tanaka S Maki M Yachi Y Asumi M. Cardiorespiratory fitness as a quantitative predictor of all-cause mortality and cardiovascular events in healthy men and women: a meta-analysis. JAMA. (2009) 301:2024–35. doi: 10.1001/jama.2009.681 19454641

[19] Benjamin EJ Muntner P Alonso A Bittencourt MS Callaway CW Carson AP. Heart disease and stroke statistics-2019 update: A report from the american heart association. Circulation. (2019) 139:e56-e528. doi: 10.1161/CIR.0000000000000659 30700139

[20] Ehrman JK Brawner CA Al-Mallah MH Qureshi WT Blaha MJ Keteyian SJ. Cardiorespiratory fitness change and mortality risk among black and white patients: henry ford exercise testing (FIT) project. Am J Med. (2017) 130:1177–83. doi: 10.1016/j.amjmed.2017.02.036 28344150

[21] Wilson PW D’Agostino RB Levy D Belanger AM Silbershatz H Kannel WB. Prediction of coronary heart disease using risk factor categories. Circulation. (1998) 97:1837–47. doi: 10.1161/01.cir.97.18.1837 9603539

[22] Duvigneaud N Matton L Wijndaele K Deriemaeker P Lefevre J Philippaerts R. Relationship of obesity with physical activity, aerobic fitness and muscle strength in Flemish adults. J Sports Med Phys Fitness (2008) 48:201–10. doi: 10.1016/j.ijms.2008.01.003 18427416

[23] Suwała S Junik R. Body mass index and waist circumference as predictors of above-average increased cardiovascular risk assessed by the SCORE2 and SCORE2-OP calculators and the proposition of new optimal cut-off values: cross-sectional single-center study. J Clin Med. (2024) 13:1931. doi: 10.3390/jcm13071931 38610696 PMC11012561

[24] Prillaman M. Why BMI is flawed - and how to redefine obesity. Nature. (2023) 622:232–3. doi: 10.1038/d41586-023-03143-x 37903933

[25] Ross R Neeland IJ Yamashita S Shai I Seidell J Magni P. Waist circumference as a vital sign in clinical practice: a Consensus Statement from the IAS and ICCR Working Group on Visceral Obesity. Nat Rev Endocrinol. (2020) 16:177–89. doi: 10.1038/s41574-019-0310-7 PMC7027970 32020062

[26] Jun JE Kang M Jin S-M Kim K Hwang Y-C Jeong I-K. Additive effect of low skeletal muscle mass and abdominal obesity on coronary artery calcification. Eur J Endocrinol. (2021) 184:867–77. doi: 10.1530/EJE-20-0885 33852417

[27] Wang S Shi S Huang Y Huang H Zhong VW. Severity of abdominal obesity and cardiometabolic diseases in US adults. Public Health. (2024) 227:154–62. doi: 10.1016/j.puhe.2023.12.010 38232563

[28] Fogelholm M Malmberg J Suni J Santtila M Kyröläinen H Mäntysaari M. Waist circumference and BMI are independently associated with the variation of cardio-respiratory and neuromuscular fitness in young adult men. Int J Obes (Lond). (2006) 30:962–9. doi: 10.1038/sj.ijo.0803243 16432537

[29] American Diabetes Association. Diagnosis and classification of diabetes mellitus. Diabetes Care. (2014) 37 Suppl 1:S81–90. doi: 10.2337/dc14-S081 24357215

[30] Ahmad E Lim S Lamptey R Webb DR Davies MJ. Type 2 diabetes. Lancet. (2022) 400:1803–20. doi: 10.1016/S0140-6736(22)01655-5 36332637

[31] Moebus S Göres L Lösch C Jöckel KH. Impact of time since last caloric intake on blood glucose levels. Eur J Epidemiol. (2011) 26:719–28. doi: 10.1007/s10654-011-9608-z PMC3186886 21822717

[32] American Diabetes Association. Standards of medical care in diabetes–2014. Diabetes Care. (2014) 37 Suppl 1:S14–80. doi: 10.2337/dc14-S014 24357209

[33] Alvares TS Souza LVM DE Soares RN Lessard SJ. Cardiorespiratory fitness is impaired in type 1 and type 2 diabetes: A systematic review, meta-analysis, and meta-regression. Med Sci Sports Exerc. (2024) 56:1553–62. doi: 10.1249/MSS.0000000000003451 PMC11327000 38650120

[34] Abushamat LA McClatchey PM Scalzo RL Schauer I Huebschmann AG Nadeau KJ. Mechanistic causes of reduced cardiorespiratory fitness in type 2 diabetes. J Endocr Soc. (2020) 4:bvaa063. doi: 10.1210/jendso/bvaa063 32666009 PMC7334033

[35] Barrett EJ Wang H Upchurch CT Liu Z. Insulin regulates its own delivery to skeletal muscle by feed-forward actions on the vasculature. Am J Physiol Endocrinol Metab. (2011) 301:E252–263. doi: 10.1152/ajpendo.00186.2011 PMC3154531 21610226

[36] Liu Z Liu J Jahn LA Fowler DE Barrett EJ. Infusing lipid raises plasma free fatty acids and induces insulin resistance in muscle microvasculature. J Clin Endocrinol Metab. (2009) 94:3543–9. doi: 10.1210/jc.2009-0027 PMC2741712 19567533

[37] Kusters YHAM Barrett EJ. Muscle microvasculature’s structural and functional specializations facilitate muscle metabolism. Am J Physiol Endocrinol Metab. (2016) 310:E379–387. doi: 10.1152/ajpendo.00443.2015 PMC4888529 26714849

[38] Baron AD Tarshoby M Hook G Lazaridis EN Cronin J Johnson A. Interaction between insulin sensitivity and muscle perfusion on glucose uptake in human skeletal muscle: evidence for capillary recruitment. Diabetes. (2000) 49:768–74. doi: 10.2337/diabetes.49.5.768 10905485

[39] Clerk LH Vincent MA Barrett EJ Lankford MF Lindner JR. Skeletal muscle capillary responses to insulin are abnormal in late-stage diabetes and are restored by angiotensin-converting enzyme inhibition. Am J Physiol Endocrinol Metab. (2007) 293:E1804–1809. doi: 10.1152/ajpendo.00498.2007 17911341

[40] McClatchey PM Williams IM Xu Z Mignemi NA Hughey CC McGuinness OP. Perfusion controls muscle glucose uptake by altering the rate of glucose dispersion *in vivo* . Am J Physiol Endocrinol Metab. (2019) 317:E1022–36. doi: 10.1152/ajpendo.00260.2019 PMC6957378 31526289

[41] Vlassara H Uribarri J. Advanced glycation end products (AGE) and diabetes: cause, effect, or both? Curr Diabetes Rep. (2014) 14:453. doi: 10.1007/s11892-013-0453-1 PMC3903318 24292971

[42] Yan SF Ramasamy R Schmidt AM. The RAGE axis: a fundamental mechanism signaling danger to the vulnerable vasculature. Circ Res. (2010) 106:842–53. doi: 10.1161/CIRCRESAHA.109.212217 PMC2862596 20299674

[43] Huebschmann AG Regensteiner JG Vlassara H Reusch JEB. Diabetes and advanced glycoxidation end products. Diabetes Care. (2006) 29:1420–32. doi: 10.2337/dc05-2096 16732039

[44] Avogaro A Fadini GP Gallo A Pagnin E de Kreutzenberg S. Endothelial dysfunction in type 2 diabetes mellitus. Nutr Metab Cardiovasc Dis. (2006) 16 Suppl 1:S39–45. doi: 10.1016/j.numecd.2005.10.015 16530129

[45] Toth PP Barter PJ Rosenson RS Boden WE Chapman MJ Cuchel M. High-density lipoproteins: a consensus statement from the National Lipid Association. J Clin Lipidol. (2013) 7:484–525. doi: 10.1016/j.jacl.2013.08.001 24079290

[46] Liu Y Zhu J Yu J Zhang X. Cardiorespiratory fitness and metabolic risk in Chinese population: evidence from a prospective cohort study. BMC Public Health. (2024) 24:522. doi: 10.1186/s12889-024-17742-4 38378502 PMC10877742

[47] Musa DI Toriola AL Abubakar NO Omachi S Olowoleni VB Ayodele KB. Association of adiposity and fitness with triglyceride-to-high-density lipoprotein cholesterol ratio in youth. Ann Pediatr Cardiol. (2023) 16:194–200. doi: 10.4103/apc.apc_1_23 37876951 PMC10593276

[48] Gordon DJ Probstfield JL Garrison RJ Neaton JD Castelli WP Knoke JD. High-density lipoprotein cholesterol and cardiovascular disease. Four prospective Am Stud Circ. (1989) 79:8–15. doi: 10.1161/01.cir.79.1.8 2642759

[49] Emerging Risk Factors Collaboration. Di Angelantonio E, Sarwar N, Perry P, Kaptoge S, Ray KK, Thompson A, Wood AM, Lewington S, Sattar N, et al. Major lipids, apolipoproteins, and risk of vascular disease. JAMA. (2009) 302:1993–2000. doi: 10.1001/jama.2009.1619 19903920 PMC3284229

[50] Madsen CM Varbo A Nordestgaard BG. Extreme high high-density lipoprotein cholesterol is paradoxically associated with high mortality in men and women: two prospective cohort studies. Eur Heart J. (2017) 38:2478–86. doi: 10.1093/eurheartj/ehx163 28419274

[51] Liu C Dhindsa D Almuwaqqat Z Ko Y-A Mehta A Alkhoder AA. Association between high-density lipoprotein cholesterol levels and adverse cardiovascular outcomes in high-risk populations. JAMA Cardiol. (2022) 7:672–80. doi: 10.1001/jamacardio.2022.0912 PMC9118072 35583863

[52] Investigators AIM-HIGH Boden WE Probstfield JL Anderson T Chaitman BR Desvignes-Nickens P. Niacin in patients with low HDL cholesterol levels receiving intensive statin therapy. N Engl J Med. (2011) 365:2255–67. doi: 10.1056/NEJMoa1107579 22085343

[53] Lincoff AM Nicholls SJ Riesmeyer JS Barter PJ Brewer HB Fox KAA. Evacetrapib and cardiovascular outcomes in high-risk vascular disease. N Engl J Med. (2017) 376:1933–42. doi: 10.1056/NEJMoa1609581 28514624

[54] Mendelsohn ME Karas RH. The protective effects of estrogen on the cardiovascular system. N Engl J Med. (1999) 340:1801–11. doi: 10.1056/NEJM199906103402306 10362825

[55] Karpe F Pinnick KE. Biology of upper-body and lower-body adipose tissue–link to whole-body phenotypes. Nat Rev Endocrinol. (2015) 11:90–100. doi: 10.1038/nrendo.2014.185 25365922

[56] Kontush A Lindahl M Lhomme M Calabresi L Chapman MJ Davidson WS. Structure of HDL: particle subclasses and molecular components. Handb Exp Pharmacol. (2015) 224:3–51. doi: 10.1007/978-3-319-09665-0_1 25522985

[57] Sloan RA. Estimated cardiorespiratory fitness and metabolic risks. Int J Environ Res Public Health. (2024) 21:635. doi: 10.3390/ijerph21050635 38791849 PMC11120962

[58] Ross R Blair SN Arena R Church TS Després J-P Franklin BA. Importance of assessing cardiorespiratory fitness in clinical practice: A case for fitness as a clinical vital sign: A scientific statement from the american heart association. Circulation. (2016) 134:e653–99. doi: 10.1161/CIR.0000000000000461 27881567

[59] Hermansen L Saltin B. Oxygen uptake during maximal treadmill and bicycle exercise. J Appl Physiol. (1969) 26:31–7. doi: 10.1152/jappl.1969.26.1.31 5762873

