# Supplementary material for: Association between metabolic health indicators and cardiorespiratory fitness in urban young and middle-aged population: a retrospective cross-sectional study
Source: Front Endocrinol (Lausanne). 2025 May 21;16:1489152. doi: 10.3389/fendo.2025.1489152 (PMC12133495; doi:10.3389/fendo.2025.1489152)
Supplement: Supplementary file 1 [file DataSheet1.docx]

Supplementary Material

# 1. Supplementary Figures


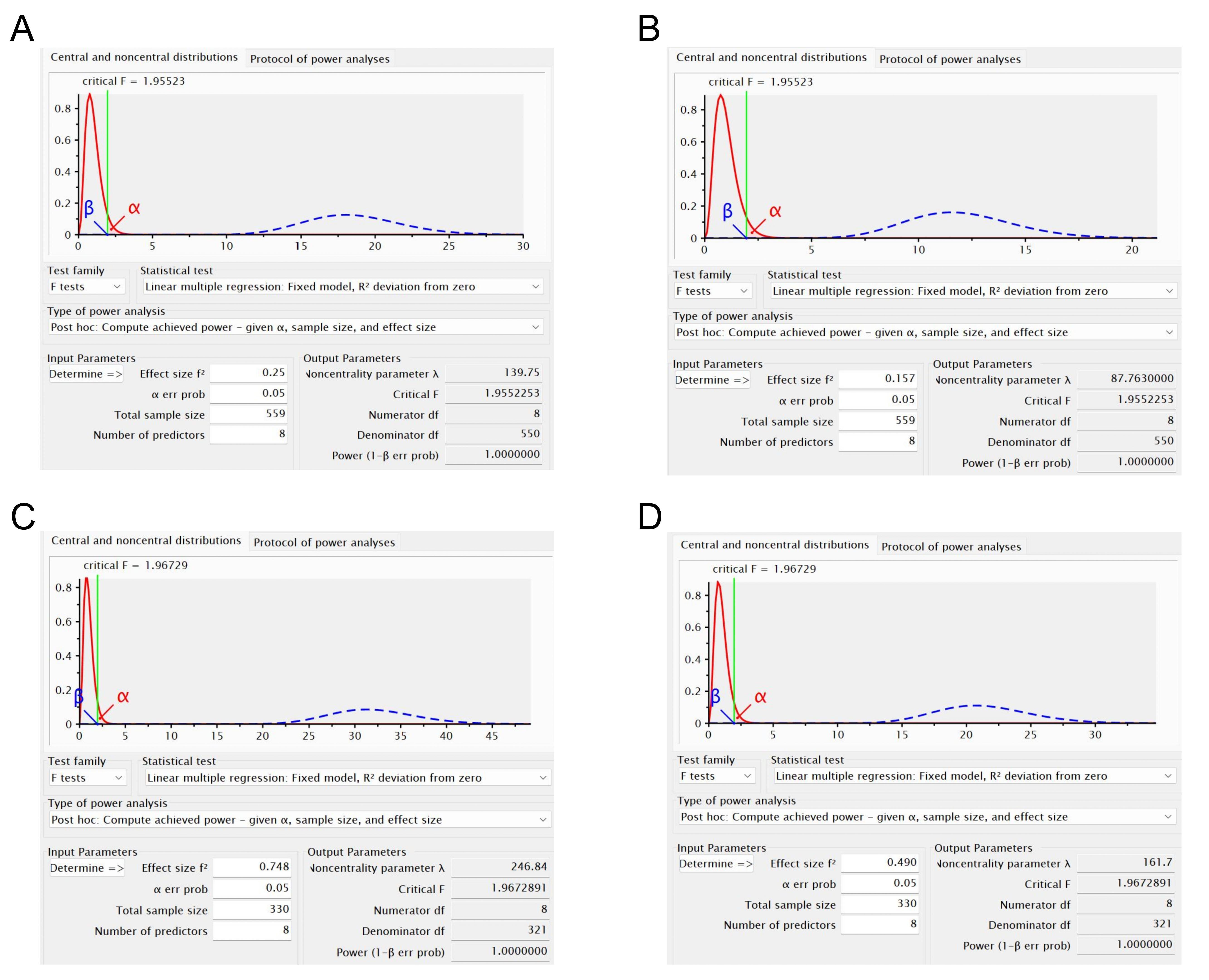
**Supplementary Figure 1.** Post-hoc power analysis of the multiple linear regression model. A, male/VO_2peak_; B, male/AT; C, female/VO_2peak_; D, female/AT.


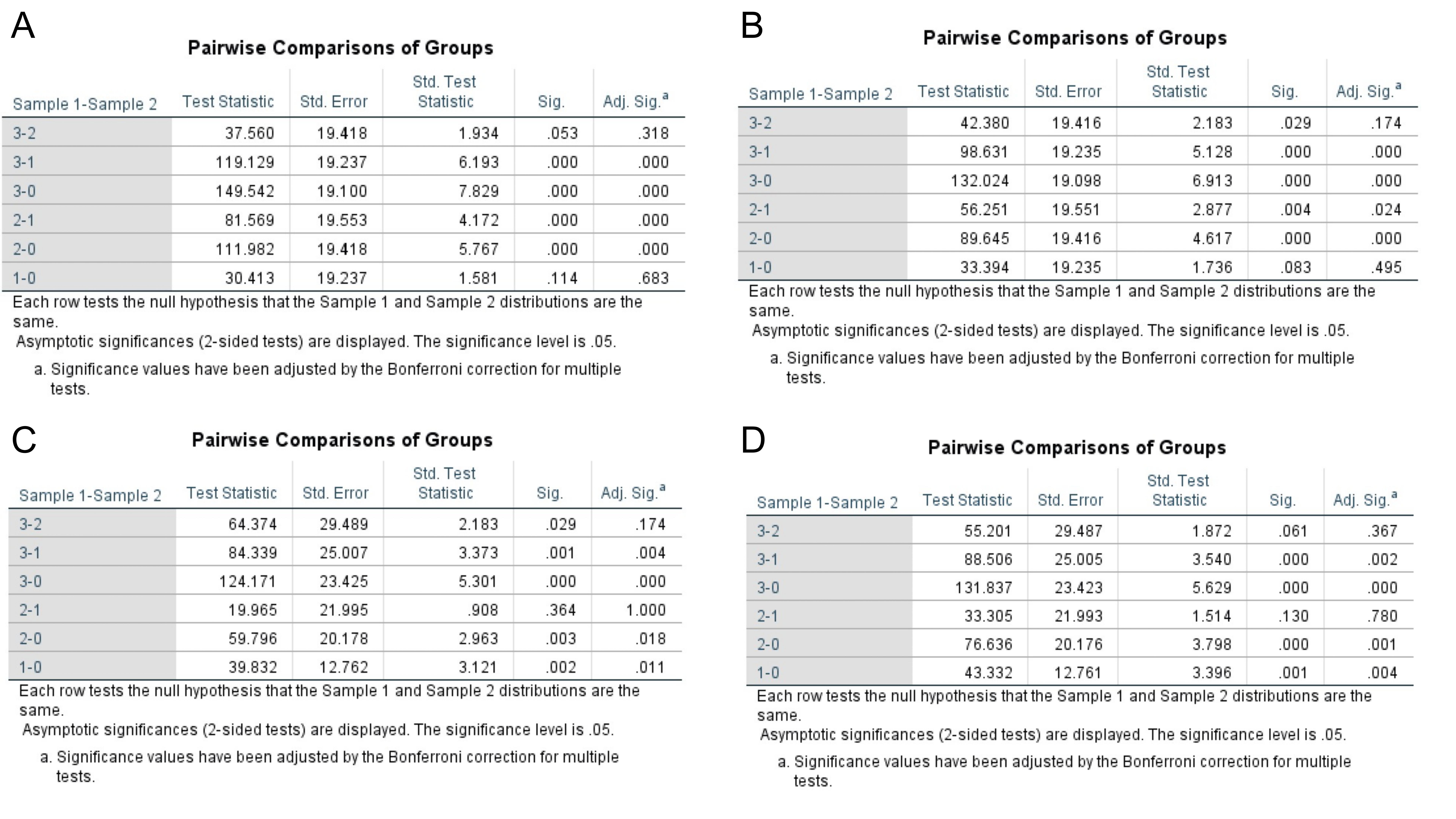
**Supplementary Figure 2.** Pairwise comparisons of groups. A, male/VO_2peak_; B, male/AT; C, female/VO_2peak_; D, female/AT. Sample 0, the number of MS components (n = 0); Sample 1, the number of MS components (n = 1); Sample 2, the number of MS components (n = 2); Sample 3, the number of MS components (n ≥ 3).

# 2. Supplementary Tables

**Supplementary Table 1.** Multicollinearity statistics and Correlations. (Metabolic health indicators were used as independent variables, and VO_2peak_ was used as dependent variables).

| **Variable** | **Model I** | | | | | | | | | |
| --- | --- | --- | --- | --- | --- | --- | --- | --- | --- | --- |
|  | **B** | **SE** | ***β* (95% CI)** | **t** | ***P*-value** | **Correlations** | | | **Multicollinearity statistics** | |
|  |  |  |  |  |  | **Zero-order** | **Partial** | **Part** | **Tolerance** | **VIF** |
| **Male (n=559)** |  |  |  |  |  |  |  |  |  |  |
| SBP (mmHg) | 0.027 | 0.018 | 0.083 (−0.007 to 0.062) | 1.552 | 0.121 | −0.113 | 0.066 | 0.060 | 0.513 | 1.950 |
| DBP (mmHg) | −0.040 | 0.024 | −0.090 (−0.087 to 0.007) | −1.666 | 0.096 | −0.180 | −0.071 | -0.064 | 0.506 | 1.975 |
| BMI (kg/m^2^) | −0.099 | 0.111 | −0.067 (−0.318 to 0.120) | −0.890 | 0.374 | −0.329 | −0.038 | -0.034 | 0.263 | 3.809 |
| WC (cm) | −0.128 | 0.043 | −0.227 (−0.212 to −0.044) | −2.986 | 0.003 | −0.370 | −0.126 | -0.115 | 0.256 | 3.912 |
| FPG (mmol/L) | −0.798 | 0.199 | −0.161 (−1.189 to −0.406) | −4.000 | ＜0.001 | −0.224 | −0.168 | -0.154 | 0.920 | 1.087 |
| TC (mmol/L) | −0.200 | 0.204 | −0.042 (−0.601 to 0.201) | −0.978 | 0.328 | −0.091 | −0.042 | -0.038 | 0.802 | 1.247 |
| TG (mmol/L) | −0.152 | 0.102 | −0.065 (−0.352 to 0.048) | −1.489 | 0.137 | −0.165 | −0.063 | -0.057 | 0.784 | 1.275 |
| HDL-C (mmol/L) | 1.538 | 0.637 | 0.108 (0.287 to 2.790) | 2.414 | 0.016 | 0.193 | 0.102 | 0.093 | 0.743 | 1.346 |
| **Female (n=330)** |  |  |  |  |  |  |  |  |  |  |
| SBP (mmHg) | 0.035 | 0.019 | 0.115 (−0.004 to 0.073) | 1.782 | 0.076 | −0.218 | 0.099 | 0.075 | 0.432 | 2.317 |
| DBP (mmHg) | −0.016 | 0.025 | −0.040 (−0.066 to 0.033) | −0.649 | 0.517 | −0.224 | −0.036 | −0.027 | 0.460 | 2.176 |
| BMI (kg/m^2^) | −0.004 | 0.106 | −0.004 (−0.213 to 0.205) | −0.036 | 0.971 | −0.566 | −0.002 | −0.002 | 0.181 | 5.533 |
| WC (cm) | −0.255 | 0.043 | −0.592 (−0.340 to −0.171) | −5.932 | < 0.001 | −0.626 | −0.314 | −0.251 | 0.180 | 5.568 |
| FPG (mmol/L) | −1.345 | 0.389 | −0.181 (−2.110 to −0.580) | −3.459 | 0.001 | −0.458 | −0.190 | −0.146 | 0.654 | 1.528 |
| TC (mmol/L) | 0.097 | 0.253 | 0.018 (−0.402 to 0.596) | 0.382 | 0.702 | 0.026 | 0.021 | 0.016 | 0.848 | 1.180 |
| TG (mmol/L) | 0.288 | 0.371 | 0.041 (−0.441 to 1.018) | 0.778 | 0.437 | −0.169 | 0.043 | 0.033 | 0.656 | 1.524 |
| HDL-C (mmol/L) | −0.346 | 0.653 | −0.029 (−1.631 to 0.939) | −0.529 | 0.597 | 0.269 | −0.030 | −0.022 | 0.614 | 1.628 |

| **Variable** | **Model II** | | | | | | | | | |
| --- | --- | --- | --- | --- | --- | --- | --- | --- | --- | --- |
|  | **B** | **SE** | ***β* (95% CI)** | **t** | ***P*-value** | **Correlations** | | | **Multicollinearity statistics** | |
|  |  |  |  |  |  | **Zero-order** | **Partial** | **Part** | **Tolerance** | **VIF** |
| **Male (n=559)** |  |  |  |  |  |  |  |  |  |  |
| SBP (mmHg) | 0..022 | 0.017 | 0.067 (−0.012 to 0.056) | 1.252 | 0.211 | −0.113 | 0.053 | 0.048 | 0.508 | 1.967 |
| DBP (mmHg) | −0.029 | 0.024 | −0.065 (−0.076 to 0.019) | −1.195 | 0.233 | −0.180 | −0.051 | −0.046 | 0.496 | 2.016 |
| BMI (kg/m^2^) | −0.132 | 0.111 | 0.089 (−0.350 to 0.086) | −1.192 | 0.234 | −0.329 | −0.051 | −0.046 | 0.260 | 3.841 |
| WC (cm) | −0.111 | 0.043 | −0.196 (−0.195 to −0.026) | −2.582 | 0.010 | −0.370 | −0.110 | −0.099 | 0.252 | 3.973 |
| FPG (mmol/L) | −0.709 | 0.200 | −0.143 (−1.101 to −0.317) | −3.554 | ＜0.001 | −0.224 | −0.150 | −0.136 | 0.903 | 1.107 |
| TC (mmol/L) | −0.199 | 0.202 | −0.042 (−0.597 to 0.199) | −0.983 | 0.326 | −0.091 | −0.042 | −0.038 | 0.802 | 1.247 |
| TG (mmol/L) | −0.149 | 0.101 | −0.064 (−0.347 to 0.050) | −1.473 | 0.141 | −0.165 | −0.063 | −0.056 | 0.784 | 1.275 |
| HDL-C (mmol/L) | 1.789 | 0.636 | 0.125 (0.539 to 3.039) | 2.812 | 0.005 | 0.193 | 0.119 | 0.107 | 0.732 | 1.365 |
| **Female (n=330)** |  |  |  |  |  |  |  |  |  |  |
| SBP (mmHg) | 0.035 | 0.019 | 0.115 (−0.004 to 0.073) | 1.783 | 0.076 | −0.218 | 0.099 | 0.075 | 0.432 | 2.317 |
| DBP (mmHg) | −0.015 | 0.025 | −0.036 (−0.064 to 0.035) | −0.584 | 0.559 | −0.224 | −0.033 | −0.025 | 0.458 | 2.182 |
| BMI (kg/m^2^) | −0.022 | 0.107 | −0.020 (−0.233 to 0.189) | −0.203 | 0.839 | −0.566 | −0.011 | −0.009 | 0.177 | 5.648 |
| WC (cm) | −0.249 | 0.043 | −0.577 (−0.334 to −0.164) | −5.738 | < 0.001 | −0.626 | −0.305 | −0.243 | 0.177 | 5.659 |
| FPG (mmol/L) | −1.243 | 0.398 | −0.167 (−2.026 to −0.459) | −3.120 | 0.002 | −0.458 | −0.172 | −0.132 | 0.623 | 1.606 |
| TC (mmol/L) | 0.121 | 0.254 | 0.022 (−0.379 to 0.621) | 0.477 | 0.633 | 0.026 | 0.027 | 0.020 | 0.842 | 1.188 |
| TG (mmol/L) | 0.366 | 0.376 | 0.052 (−0.375 to 1.106) | 0.972 | 0.332 | −0.169 | 0.054 | 0.041 | 0.636 | 1.573 |
| HDL-C (mmol/L) | −0.214 | 0.662 | −0.018 (−1.517 to 1.089) | −0.323 | 0.747 | 0.269 | −0.018 | −0.014 | 0.596 | 1.677 |

Model I, crude model; Model II, adjusted for age, smoking status, and drinking status; B, unstandardized coefficient; SE, standard error; *β*, standardized regression coefficient; SBP, systolic blood pressure; DBP, diastolic blood pressure; BMI, body mass index; WC, waist circumference; FPG, fasting plasma glucose; TC, total cholesterol; TG, triglyceride; HDL-C, high-density lipoprotein cholesterol; VO_2peak_, peak oxygen uptake; AT, anaerobic threshold; VIF, variance inflation factor.

**Supplementary Table 2.** Multicollinearity statistics and Correlations. (Metabolic health indicators were used as independent variables, and AT was used as dependent variables).

| **Variable** | **Model I** | | | | | | | | | |
| --- | --- | --- | --- | --- | --- | --- | --- | --- | --- | --- |
|  | **B** | **SE** | ***β* (95% CI)** | **t** | ***P*-value** | **Correlations** | | | **Multicollinearity statistics** | |
|  |  |  |  |  |  | **Zero-order** | **Partial** | **Part** | **Tolerance** | **VIF** |
| **Male (n=559)** |  |  |  |  |  |  |  |  |  |  |
| SBP (mmHg) | 0.014 | 0.010 | 0.077 (−0.006 to 0.034) | 1.382 | 0.167 | −0.083 | 0.059 | 0.055 | 0.513 | 1.950 |
| DBP (mmHg) | −0.015 | 0.014 | −0.059 (−0.043 to 0.013) | −1.049 | 0.295 | −0.136 | −0.045 | −0.042 | 0.506 | 1.975 |
| BMI (kg/m^2^) | −0.063 | 0.065 | −0.075 (−0.191 to 0.065) | −0.964 | 0.336 | −0.296 | −0.041 | −0.038 | 0.263 | 3.809 |
| WC (cm) | −0.073 | 0.025 | −0.227 (−0.122 to −0.023) | −2.888 | 0.004 | −0.333 | −0.122 | −0.115 | 0.256 | 3.912 |
| FPG (mmol/L) | −0.397 | 0.117 | −0.140 (−0.627 to −0.167) | −3.394 | 0.001 | −0.197 | −0.143 | −0.135 | 0.920 | 1.087 |
| TC (mmol/L) | −0.009 | 0.120 | −0.003 (−0.245 to 0.226) | −0.077 | 0.939 | −0.046 | −0.003 | −0.003 | 0.802 | 1.247 |
| TG (mmol/L) | −0.009 | 0.060 | −0.007 (−0.127 to 0.108) | −0.157 | 0.876 | −0.078 | −0.007 | −0.006 | 0.784 | 1.275 |
| HDL-C (mmol/L) | 0.421 | 0.374 | 0.052 (−0.314 to 1.156) | 1.125 | 0.261 | 0.126 | 0.048 | 0.045 | 0.743 | 1.346 |
| **Female (n=330)** |  |  |  |  |  |  |  |  |  |  |
| SBP (mmHg) | 0.016 | 0.014 | 0.077 (−0.012 to 0.044) | 1.110 | 0.268 | −0.276 | 0.062 | 0.051 | 0.432 | 2.317 |
| DBP (mmHg) | −0.034 | 0.018 | −0.123 (−0.070 to 0.003) | −1.823 | 0.069 | −0.297 | −0.101 | −0.083 | 0.460 | 2.176 |
| BMI (kg/m^2^) | −0.056 | 0.078 | −0.078 (−0.210 to 0.097) | −0.721 | 0.471 | −0.505 | −0.040 | −0.033 | 0.181 | 5.533 |
| WC (cm) | −0.124 | 0.032 | −0.423 (−0.186 to −0.062) | −3.916 | < 0.001 | −0.538 | −0.214 | −0.179 | 0.180 | 5.568 |
| FPG (mmol/L) | −0.687 | 0.286 | −0.136 (−1.250 to −0.124) | −2.400 | 0.017 | −0.393 | −0.133 | −0.110 | 0.654 | 1.528 |
| TC (mmol/L) | −0.260 | 0.186 | −0.069 (−0.627 to 0.107) | −1.396 | 0.164 | −0.096 | −0.078 | −0.064 | 0.848 | 1.180 |
| TG (mmol/L) | −0.093 | 0.273 | −0.019 (−0.630 to 0.443) | −0.341 | 0.733 | −0.200 | −0.019 | −0.016 | 0.656 | 1.524 |
| HDL-C (mmol/L) | −0.830 | 0.481 | −0.101 (−1.776 to 0.115) | −1.728 | 0.085 | 0.192 | −0.096 | −0.079 | 0.614 | 1.628 |

| **Variable** | **Model II** | | | | | | | | | |
| --- | --- | --- | --- | --- | --- | --- | --- | --- | --- | --- |
|  | **B** | **SE** | ***β* (95% CI)** | **t** | ***P*-value** | **Correlations** | | | **Multicollinearity statistics** | |
|  |  |  |  |  |  | **Zero-order** | **Partial** | **Part** | **Tolerance** | **VIF** |
| **Male (n=559)** |  |  |  |  |  |  |  |  |  |  |
| SBP (mmHg) | 0.015 | 0.010 | 0.082 (−0.005 to 0.036) | 1.482 | 0.139 | −0.083 | 0.063 | 0.059 | 0.508 | 1.967 |
| DBP (mmHg) | −0.017 | 0.014 | −0.068 (−0.045 to 0.011) | −1.200 | 0.231 | −0.136 | −0.051 | −0.048 | 0.496 | 2.016 |
| BMI (kg/m^2^) | −0.056 | 0.066 | −0.067 (−0.185 to 0.073) | −0.858 | 0.391 | −0.296 | −0.037 | −0.034 | 0.260 | 3.841 |
| WC (cm) | −0.076 | 0.025 | −0.238 (−0.126 to −0.026) | −3.007 | 0.003 | −0.333 | −0.127 | −0.119 | 0.252 | 3.973 |
| FPG (mmol/L) | −0.415 | 0.118 | −0.147 (−0.647 to −0.183) | −3.516 | ＜0.001 | −0.197 | −0.148 | −0.139 | 0.903 | 1.107 |
| TC (mmol/L) | −0.009 | 0.120 | −0.003 (−0.245 to 0.226) | −0.078 | 0.938 | −0.046 | −0.003 | −0.003 | 0.802 | 1.247 |
| TG (mmol/L) | −0.010 | 0.060 | −0.007 (−0.127 to 0.107) | −0.166 | 0.868 | −0.078 | −0.007 | −0.007 | 0.784 | 1.275 |
| HDL-C (mmol/L) | 0.370 | 0.377 | 0.046 (−0.370 to 1.110) | 0.982 | 0.326 | 0.126 | 0.042 | 0.039 | 0.732 | 1.365 |
| **Female (n=330)** |  |  |  |  |  |  |  |  |  |  |
| SBP (mmHg) | 0.016 | 0.014 | 0.077 (−0.012 to 0.044) | 1.110 | 0.268 | −0.276 | 0.062 | 0.051 | 0.432 | 2.317 |
| DBP (mmHg) | −0.033 | 0.018 | −0.120 (−0.069 to 0.004) | −1.766 | 0.078 | −0.297 | −0.098 | −0.081 | 0.458 | 2.182 |
| BMI (kg/m^2^) | −0.067 | 0.079 | −0.093 (−0.223 to 0.088) | −0.854 | 0.394 | −0.505 | −0.048 | −0.039 | 0.177 | 5.648 |
| WC (cm) | −0.120 | 0.032 | −0.410 (−0.183 to −0.057) | −3.758 | < 0.001 | −0.538 | −0.206 | −0.172 | 0.177 | 5.659 |
| FPG (mmol/L) | −0.624 | 0.293 | −0.123 (−1.200 to −0.047) | −2.126 | 0.034 | −0.393 | −0.118 | −0.097 | 0.623 | 1.606 |
| TC (mmol/L) | −0.245 | 0.187 | −0.065 (−0.613 to 0.123) | −1.310 | 0.191 | −0.096 | −0.073 | −0.060 | 0.842 | 1.188 |
| TG (mmol/L) | −0.045 | 0.277 | −0.009 (−0.590 to 0.500) | −0.163 | 0.870 | −0.200 | −0.009 | −0.007 | 0.636 | 1.573 |
| HDL-C (mmol/L) | −0.749 | 0.488 | −0.091 (−1.708 to 0.211) | −1.536 | 0.126 | 0.192 | −0.086 | −0.070 | 0.596 | 1.677 |

Model I, crude model; Model II, adjusted for age, smoking status, and drinking status; B, unstandardized coefficient; SE, standard error; *β*, standardized regression coefficient; SBP, systolic blood pressure; DBP, diastolic blood pressure; BMI, body mass index; WC, waist circumference; FPG, fasting plasma glucose; TC, total cholesterol; TG, triglyceride; HDL-C, high-density lipoprotein cholesterol; VO_2peak_, peak oxygen uptake; AT, anaerobic threshold; VIF, variance inflation factor.
